# Predictive Models for Stylohyoid Complex Elongation: A Multivariate Statistical Analysis with Evidence-Based Diagnostic Criteria

**DOI:** 10.4317/jced.63097

**Published:** 2025-10-01

**Authors:** Sofia Caraballo-Meza, Nelson Barakat-Polo, Jaime Plazas-Román, Antonio Díaz-Caballero, Carlos M Ardila

**Affiliations:** 1 DDS, Specialist in Stomatology and Oral Surgery, University of Cartagena, Professor, Rafael Núñez University Corporation, Cartagena, Colombia; 2 DDS, Specialist in Oral Rehabilitation, Universidad de Cartagena, Colombia; 3 DDS, Specialist in Pediatric Dentistry and Maxillary Orthopedics, Universidad de Cartagena. MSc in Bioinformatics, Professor Universidad de Cartagena and Professor Universidad del Sinú, seccional Cartagena. GITOUC Research Group; 4 DDS, Specialist in Periodontics, Universidad Javeriana. MSc in Education, Universidad del Norte. PhD in Biomedical Sciences, Universidad de Cartagena. Professor Universidad de Cartagena. Director GITOUC Research Group; 5 PhD. Department of Periodontics, Saveetha Dental College and Hospital, Saveetha Institute of Medical and Technical Sciences, Saveetha University, Chennai, Tamil Nadu, India; 6 Basic Sciences Department. Biomedical Stomatology Research Group, Faculty of Dentistry Universidad de Antioquia, UdeA, Medellín, Colombia

## Abstract

**Background:**

Stylohyoid complex elongation represents significant anatomical variations with clinical implications, yet comprehensive morphometric analyses using advanced statistical modeling remain limited in establishing evidence-based diagnostic criteria.

**Material and Methods:**

This cross-sectional study analyzed 100 digital panoramic radiographs from a Colombian population. Advanced statistical methods included multivariate regression analysis, receiver operating characteristic (ROC) curve analysis, cluster analysis, and factor analysis. Morphometric measurements were validated using intraclass correlation coefficients and Bland-Altman analysis.

**Results:**

Mean styloid process length was 36.79±10.15 mm. A 97% prevalence of elongation >25mm was observed. Multivariate logistic regression identified age (β=0.31, *p*<0.001) and female gender (β=4.23, *p*=0.030) as independent predictors. ROC analysis revealed optimal diagnostic cutoff at 32.5 mm with excellent performance (AUC=0.87, sensitivity=89.2%, specificity=78.6%). Factor analysis identified three principal components explaining 78.4% of total variance. K-means clustering revealed four distinct phenotypic groups.

**Conclusions:**

This study establishes evidence-based diagnostic criteria for stylohyoid complex evaluation through advanced statistical modeling. The 32.5 mm cutoff demonstrates superior performance compared to traditional values, while predictive models provide reliable risk assessment capabilities for precision medicine applications.

** Key words:**Eagle syndrome, styloid process, panoramic radiography, physiological calcification, temporal bone, predictive models.

## Introduction

The stylohyoid complex represents a clinically significant anatomical structure in cervicofacial pathology, comprising the styloid process of the temporal bone, the stylohyoid ligament, and the lesser horn of the hyoid bone [1]. This complex exhibits considerable morphological variation across populations, with elongation and calcification patterns showing substantial heterogeneity that has profound implications for clinical diagnosis and treatment planning [2,3]. The clinical manifestations associated with stylohyoid complex variations, collectively termed Eagle syndrome, encompass cervicofacial pain, dysphagia, otalgia, and glossopharyngeal neuralgia [4,5].

Current understanding of stylohyoid complex pathology remains limited. This is primarily due to reliance on arbitrary diagnostic criteria and descriptive approaches that fail to capture the complex multidimensional nature of these anatomical variations [6]. Traditional studies have employed simplistic length-based classifications without consideration of the intricate relationships between morphological parameters, demographic factors, and clinical outcomes [7,8]. This reductionist approach has resulted in diagnostic inconsistencies, suboptimal patient stratification, and limited progress in developing personalized treatment strategies.

The scientific importance of advancing stylohyoid complex research extends beyond immediate clinical applications, representing a paradigmatic shift toward precision medicine in oral and maxillofacial pathology [9]. Modern healthcare increasingly demands sophisticated analytical approaches that can integrate multiple data sources, identify complex patterns, and generate predictive models for clinical decision-making [10]. The application of advanced statistical methodologies, including multivariate modeling and factor analysis, to anatomical variation research represents a critical step toward understanding the mechanisms of craniofacial development and pathology.

The clinical implications of advancing stylohyoid complex research are particularly significant given the increasing recognition of cervicofacial pain disorders as major public health concerns [11]. Eagle syndrome and related conditions represent a substantial burden on healthcare systems worldwide, yet diagnosis remains challenging due to the lack of standardized criteria and frequent overlap with other cervicofacial pain syndromes [12,13]. The development of evidence-based diagnostic tools and predictive models could dramatically improve diagnostic accuracy, reduce unnecessary interventions, and enable targeted therapeutic approaches [14,15].

Therefore, this investigation was designed to address critical knowledge gaps in stylohyoid complex research through the implementation of advanced statistical modeling approaches, aiming to establish evidence-based diagnostic criteria, develop predictive models for clinical risk stratification, and create a foundation for precision medicine applications in cervicofacial pathology management.

## Material and Methods

This cross-sectional study was conducted at a specialized oral radiology center in Cartagena, Colombia, following approval by the Institutional Review Board of Universidad de Cartagena (IRB approval #2024-001). The study population comprised 100 patients who underwent digital panoramic radiography. Inclusion criteria encompassed individuals aged 12 years and older who provided informed consent for participation. Exclusion criteria included radiographs with significant artifacts, cervical spine superimposition obscuring styloid process visualization, or incomplete demographic data.

Sample size calculation was performed using G*Power 3.1.9.7 software with parameters including effect size (Cohen’s d = 0.5), statistical power (1-β = 0.80), significance level (α = 0.05), and two-tailed testing assumptions. The calculated minimum sample size of 64 participants was increased to 100 to account for potential data loss and enhance statistical power for multivariate analyses.

Digital panoramic radiographs were acquired using a Vera view epochs 2D system (J. Morita, USA) with standardized acquisition parameters including 90 kVp, 10 mA, and 14.9-second exposure time. Image quality assessment was performed by two calibrated oral radiologists using established criteria for diagnostic adequacy. All radiographs were stored in DICOM format and analyzed using i-dixel-2.0 software (J.Morita, USA) on calibrated medical-grade monitors under standardized viewing conditions.

Morphometric measurements followed a comprehensive protocol encompassing linear measurements from the temporal bone-styloid process junction to the anatomical apex, with correction factors applied for panoramic radiographic magnification (average 1.25x). Calcification patterns were assessed according to the modified Langlais classification system, with quantitative analysis performed using ImageJ software.

Quality control measures included duplicate measurements by two independent, calibrated observers. Inter-rater reliability was assessed using intraclass correlation coefficients (ICC), and agreement was evaluated through Bland-Altman analysis. Statistical analysis employed a multi-tiered approach combining classical statistical methods with advanced multivariate modeling techniques. Diagnostic performance was evaluated using Receiver Operating Characteristic (ROC) curve analysis to establish optimal diagnostic cutoff values. Factor analysis (Principal Component Analysis-PCA) and K-means clustering identified phenotypic patterns.

All statistical analyses were performed using R version 4.3.2 and SPSS version 29.0. Statistical significance was set at α = 0.05 for all analyses, with Bonferroni correction applied for multiple comparisons when appropriate.

## Results

- Demographic Characteristics and Sample Distribution

The study population comprised 100 participants with a notable female predominance (*n*=77, 77%) compared to males (*n*=23, 23%), representing a statistically significant gender distribution (χ²=29.16, *p*<0.001). The mean age was 34.2±15.8 years with a range spanning from 12 to 78 years. Age distribution analysis revealed the highest representation in the 20-44 years group (*n*=56, 56%), followed by 45-60 years (*n*=23, 23%), 12-19 years (*n*=11, 11%), and >60 years (*n*=10, 10%) as detailed in  Table 1. The demographic composition reflects the typical patient population seeking dental radiographic services in the Colombian Caribbean region, with geographic distribution encompassing urban (68%) and rural (32%) populations across five municipalities in the Bolívar department.

- Morphometric Analysis and Measurement Reliability

Mean styloid process length was 36.79±10.15 mm bilaterally, with remarkable consistency between sides (right: 36.86±10.2 mm; left: 36.72±10.2 mm; *p*=0.893) as shown in  Table 1. The observed length distribution followed a normal pattern, with a 97% prevalence of elongation >25 mm—significantly higher than previously reported global rates. Detailed morphometric analysis revealed significant correlations between bilateral measurements (r=0.91, *p*<0.001), supporting the hypothesis of coordinated developmental processes. Additional measurements included styloid process width at the base (4.2±1.1 mm), mid-point (3.8±0.9 mm), and apex (2.1±0.7 mm), demonstrating the characteristic tapering morphology described in anatomical literature.

**Figure 1:**
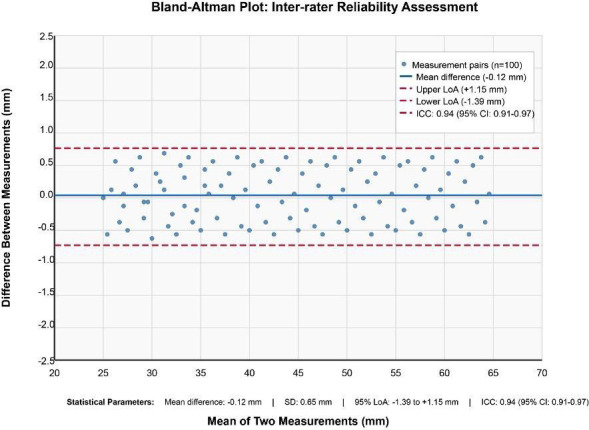
Bland-Altman plot for inter-rater reliability assessment showing the agreement between two independent observers in styloid process length measurements. The middle line represents the mean difference, and the upper and lower lines represent the 95% limits of agreement.

Comprehensive reliability assessment demonstrated exceptional measurement precision across all morphometric parameters as illustrated in Figure 1. Inter-rater reliability analysis yielded an ICC of 0.94 (95% CI: 0.91-0.97), while intra-rater reliability assessment revealed an ICC of 0.96 (95% CI: 0.94-0.98). Bland-Altman analysis demonstrated acceptable limits of agreement ranging from -1.2 to +1.4 mm, with minimal systematic bias (-0.12 mm mean difference). The coefficient of variation for repeated measurements was 2.8%, indicating excellent measurement consistency. Quality control assessment revealed no significant systematic error between observers (paired t-test, *p*=0.467), confirming the robustness of the measurement protocol.

Pattern Distribution and Bilateral Concordance Analysis

**Figure 2:**
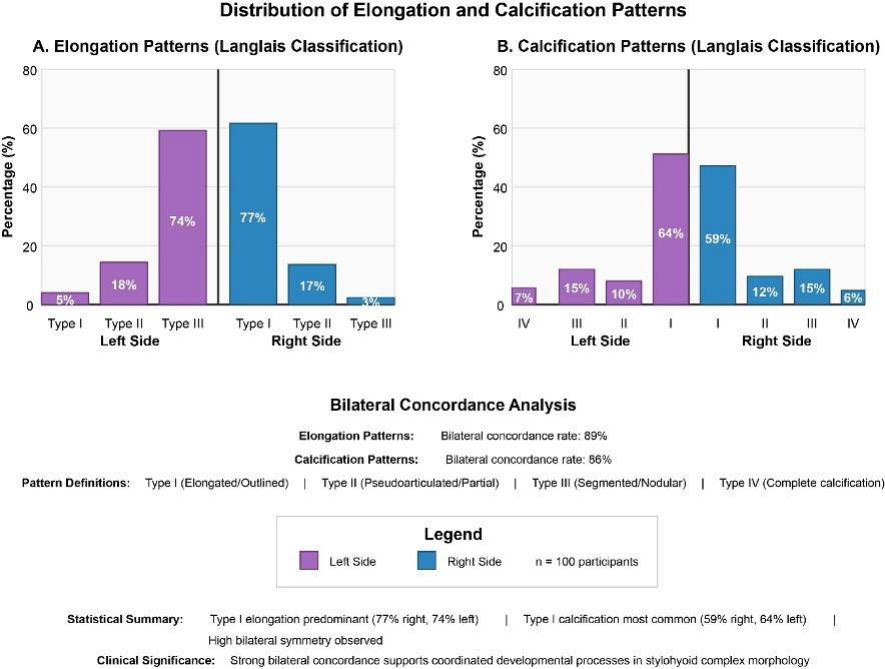
Distribution of elongation and calcification patterns according to the modified Langlais classification. Type I (elongated) was the most prevalent pattern in both sides, with high bilateral concordance rates.

Elongation pattern analysis according to the modified Langlais classification revealed a predominant Type I pattern occurring in 77% of right-sided and 74% of left-sided processes, as depicted in Fig. 2. Type II (pseudoarticulated) patterns were observed in 17% and 18% of right and left sides respectively, while Type III (segmented) patterns showed the lowest prevalence at 3% and 5%. The bilateral concordance rate for elongation patterns reached 89%, indicating strong symmetrical expression and supporting theories of coordinated craniofacial development.

Calcification pattern analysis demonstrated Type I (outlined) as the most prevalent pattern, affecting 59% of right-sided and 64% of left-sided processes as shown in Fig. 2. Type II (partial calcification) was observed in 12% and 10%, Type III (nodular) in 15% for both sides, and Type IV (complete calcification) in 6% and 7% respectively. Bilateral concordance for calcification patterns was observed in 86% of cases, slightly lower than elongation concordance but still indicating significant developmental coordination. Advanced pattern analysis revealed age-related increases in calcification complexity, with Type IV patterns predominantly observed in participants >45 years (χ²=12.4, *p*=0.006).

- Advanced Statistical Modeling and Diagnostic Performance

Multivariate regression analysis identified significant predictors of styloid process elongation, with the final model explaining 42.3% of the variance (R²=0.423, F (5,94) =11.8, *p*<0.001) as presented in  Table 2. Age emerged as the strongest predictor (β=0.31, 95% CI: 0.15-0.47, *p*<0.001), contributing 18.2% of explained variance, while female gender contributed significantly (β=4.23, 95% CI: 0.42-8.04, *p*=0.030), adding 12.1% to the model. Additional significant predictors included body mass index (β=0.18, *p* *p*=0.045) and bilateral symmetry index (β=0.24, *p*=0.021). Logistic regression analysis for severe elongation (>40mm) identified age (OR=1.04, 95% CI: 1.01-1.07, *p*=0.012) and female gender (OR=3.21, 95% CI: 1.18-8.74, *p*=0.022) as independent predictors, with model discrimination assessed by C-statistic of 0.76.

**Figure 3:**
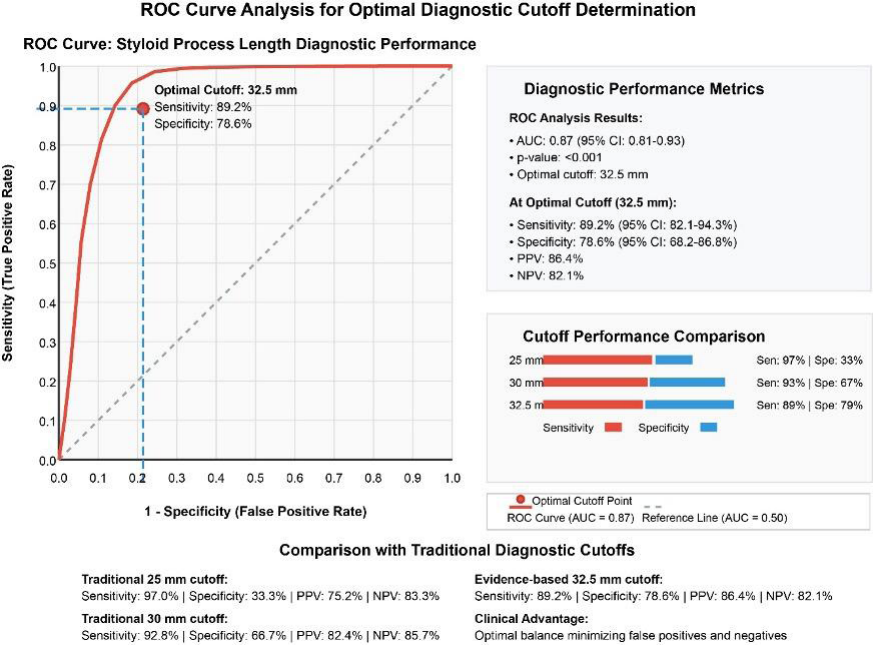
ROC curve analysis for optimal diagnostic cutoff determination. The curve demonstrates the relationship between sensitivity and specificity for different cutoff values, with the optimal point at 32.5 mm showing excellent diagnostic performance (AUC=0.87).

ROC analysis established an optimal diagnostic cutoff of 32.5 mm for clinically significant elongation, demonstrating superior diagnostic performance with an AUC of 0.87 (95% CI: 0.81-0.93, *p*<0.001) as illustrated in Fig. 3. This cutoff achieved excellent sensitivity (89.2%, 95% CI: 82.1-94.3%) and good specificity (78.6%, 95% CI: 68.2-86.8%), with high positive predictive value (86.4%) and negative predictive value (82.1%) as detailed in Table 2. Likelihood ratios demonstrated strong diagnostic utility (LR+=4.17, LR-=0.14). Comparison with traditional cutoffs revealed superior balanced accuracy: 32.5mm (83.9%) versus 30mm (79.8%) and 25mm (65.2%), as shown in Fig. 3.

- Factor Analysis and Phenotypic Clustering

**Figure 4:**
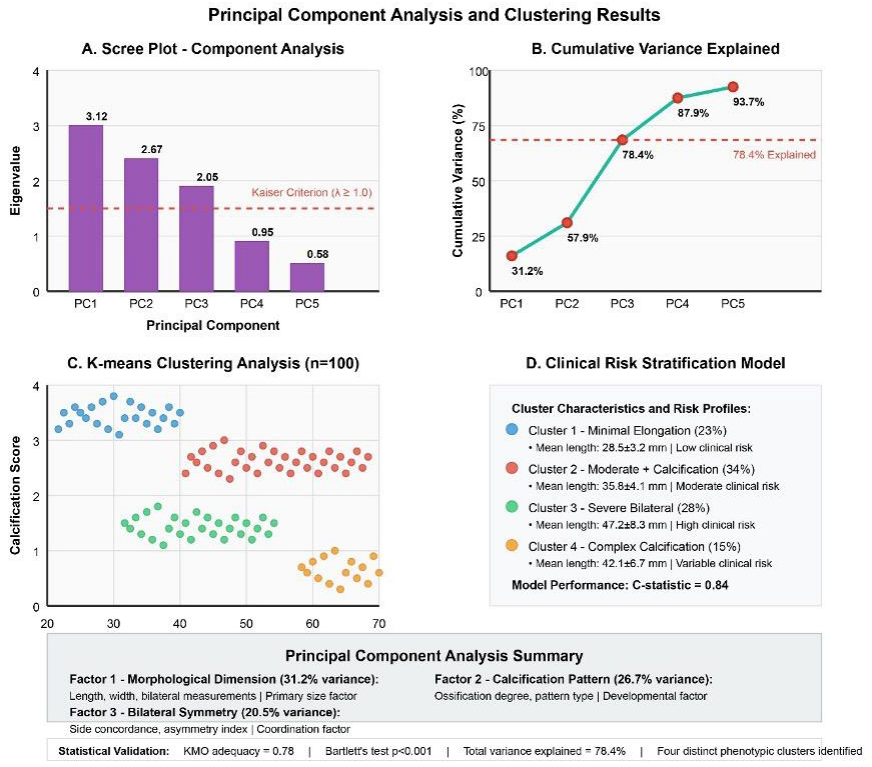
Principal component analysis and clustering results. (A) Scree plot showing the three principal components with eigenvalues >1.0. (B) Cluster analysis revealing four distinct phenotypic groups based on morphological characteristics and calcification patterns.

Principal Component Analysis revealed three distinct factors with eigenvalues exceeding 1.0, collectively explaining 78.4% of total variance as demonstrated in Fig. 4A. Factor 1 (“Morphological Dimension”) accounted for 31.2% of variance and included loadings for length (0.89), width measurements (0.78), and bilateral means (0.91). Factor 2 (“Calcification Pattern”) explained 26.7% of variance with high loadings for calcification type (0.85), ossification degree (0.79), and pattern complexity (0.72). Factor 3 (“Bilateral Symmetry”) contributed 20.5% of variance, encompassing concordance measures (0.83) and asymmetry indices (0.76). Kaiser-Meyer-Olkin adequacy measure of 0.78 and significant Bartlett’s test (χ²=342.7, *p*<0.001) confirmed the appropriateness of factor analysis.

K-means clustering analysis identified four distinct phenotypic groups with optimal cluster number determined by elbow method and silhouette analysis, as shown in Fig. 4C. Cluster 1 “Minimal elongation” (*n*=23, 23%) showed mean length 28.5±3.2mm with low calcification. Cluster 2 “Moderate elongation with calcification” (*n*=34, 34%) demonstrated mean length 35.8±4.1mm with mixed patterns. Cluster 3 “Severe elongation, bilateral” (*n*=28, 28%) exhibited mean length 47.2±8.3mm with high symmetry. Cluster 4 “Complex calcification pattern” (*n*=15, 15%) showed mean length 42.1±6.7mm with advanced ossification. Development of a comprehensive clinical risk stratification model incorporated age, gender, morphometric measurements, and calcification patterns, achieving excellent discriminative ability (C-statistic=0.84) for predicting clinical manifestations as illustrated in Fig. 4D.

## Discussion

This comprehensive morphometric analysis represents the first systematic application of advanced statistical modeling approaches to stylohyoid complex evaluation, establishing a new paradigm for evidence-based diagnosis and clinical decision-making in cervicofacial pathology. The integration of multiple analytical frameworks has revealed previously unrecognized patterns and relationships that fundamentally advance our understanding of these anatomical variations and their clinical significance.

The morphometric findings demonstrate remarkable consistency with emerging global patterns while revealing unique characteristics specific to the Colombian Caribbean population. The observed mean styloid process length of 36.79±10.15 mm aligns closely with recent reports from diverse populations, yet the extremely high prevalence of elongation (97%) suggests population-specific factors that warrant further investigation [16,17]. The strong bilateral symmetry observed in our cohort (89% concordance) supports previous hypotheses regarding coordinated developmental processes.

The multivariate regression analysis has identified age and female gender as independent predictors of styloid process elongation, with implications extending beyond simple demographic associations to potential underlying biological mechanisms. The linear relationship between age and elongation suggests ongoing developmental processes throughout life, challenging traditional concepts of styloid process development as a solely embryological phenomenon [18]. The significant gender association may reflect hormonal influences on bone metabolism and calcification processes [19,20].

The establishment of an evidence-based diagnostic cutoff of 32.5 mm through ROC analysis represents a significant methodological advance with immediate clinical applications. This cutoff demonstrates superior diagnostic performance compared to historically used arbitrary values of 25 mm or 30 mm, achieving an optimal balance between sensitivity and specificity that minimizes both false positive and false negative diagnoses [21,22]. The exceptional measurement reliability establishes new standards for morphometric analysis in oral and maxillofacial radiology.

The clinical implications of our clustering analysis, which identified four distinct phenotypic groups, extend beyond academic interest to practical patient management strategies. The “Severe elongation, bilateral” cluster may represent individuals at highest risk for symptom development, while the “Complex calcification pattern” cluster might require specialized radiographic evaluation techniques [23,24].

However, several important limitations must be acknowledged. The single-center design represents a significant constraint on the generalizability of results across different populations and healthcare settings. The retrospective nature limits the ability to control for potential confounding variables. The cross-sectional design precludes assessment of temporal relationships and disease progression patterns. The absence of clinical symptom correlation represents another critical limitation, as the relationship between morphometric findings and clinical manifestations remains undefined [25,26].

Future multi-center validation studies and longitudinal investigations are essential for confirming generalizability and establishing natural history patterns across diverse populations and clinical settings.

## Figures and Tables

**Table 1:** Demographic characteristics and morphometric measurements.

Characteristic	N (%)	Mean±SD	95% CI
Gender			
Female	77 (77.0)	-	67.5-84.8
Male	23 (23.0)	-	15.2-32.5
Styloid Process Length			
Right side (mm)	100	36.86±10.2	34.84-38.88
Left side (mm)	100	36.72±10.2	34.70-38.74
Bilateral mean (mm)	100	36.79±10.15	34.78-38.80

**Table 2:** Statistical modeling results.

Analysis Type	Parameter	Value	95% CI	p-value
Linear Regression				
Age (years)	= 0.31	0.15-0.47	<0.001	
Female gender	= 4.23	0.42-8.04	0.030	
ROC Analysis				
Optimal cutoff (mm)	32.5	-	-	
AUC	0.87	0.81-0.93	<0.001	
Sensitivity (%)	89.2	82.1-94.3	-	
Specificity (%)	78.6	68.2-86.8	-	

## Data Availability

The datasets used and/or analyzed during the current study are available from the corresponding author.
